# Transcranial Direct Current Stimulation on Parkinson's Disease: Systematic Review and Meta-Analysis

**DOI:** 10.3389/fneur.2021.794784

**Published:** 2022-01-10

**Authors:** Paloma Cristina Alves de Oliveira, Thiago Anderson Brito de Araújo, Daniel Gomes da Silva Machado, Abner Cardoso Rodrigues, Marom Bikson, Suellen Marinho Andrade, Alexandre Hideki Okano, Hougelle Simplicio, Rodrigo Pegado, Edgard Morya

**Affiliations:** ^1^Program in Neuroengineering, Edmond and Lily Safra International Institute of Neuroscience, Santos Dumont Institute, Macaíba, Brazil; ^2^Department of Physical Education, Federal University of Rio Grande do Norte, Natal, Brazil; ^3^Department of Biomedical Engineering, The City College of New York, New York, NY, United States; ^4^Neuroscience and Aging Laboratory, Federal University of Paraíba, João Pessoa, Brazil; ^5^Center for Mathematics, Computing and Cognition, Federal University of ABC, São Bernardo do Campo, Brazil; ^6^Rehabilitation Center, Anita Garibaldi Center for Education and Health, Santos Dumont Institute, Macaíba, Brazil; ^7^Department of Biomedical Sciences, State University of Rio Grande do Norte, Mossoró, Brazil; ^8^Neuron—Care Unit in Neurosurgery, Hospital Rio Grande, Natal, Brazil; ^9^Program in Rehabilitation Science, Program in Health Science, Federal University of Rio Grande do Norte, Natal, Brazil

**Keywords:** transcranial direct current stimulation (tDCS), Parkinson's disease, neuromodulation, motor symptoms, meta-analysis

## Abstract

**Background:** Clinical impact of transcranial direct current stimulation (tDCS) alone for Parkinson's disease (PD) is still a challenge. Thus, there is a need to synthesize available results, analyze methodologically and statistically, and provide evidence to guide tDCS in PD.

**Objective:** Investigate isolated tDCS effect in different brain areas and number of stimulated targets on PD motor symptoms.

**Methods:** A systematic review was carried out up to February 2021, in databases: Cochrane Library, EMBASE, PubMed/MEDLINE, Scopus, and Web of science. Full text articles evaluating effect of active tDCS (anodic or cathodic) vs. sham or control on motor symptoms of PD were included.

**Results:** Ten studies (*n* = 236) were included in meta-analysis and 25 studies (*n* = 405) in qualitative synthesis. The most frequently stimulated targets were dorsolateral prefrontal cortex and primary motor cortex. No significant effect was found among single targets on motor outcomes: Unified Parkinson's Disease Rating Scale (UPDRS) III – motor aspects (MD = −0.98%, 95% CI = −10.03 to 8.07, *p* = 0.83, *I*^2^ = 0%), UPDRS IV – dyskinesias (MD = −0.89%, CI 95% = −3.82 to 2.03, *p* = 0.55, *I*^2^ = 0%) and motor fluctuations (MD = −0.67%, CI 95% = −2.45 to 1.11, *p* = 0.46, *I*^2^ = 0%), timed up and go – gait (MD = 0.14%, CI 95% = −0.72 to 0.99, *p* = 0.75, *I*^2^ = 0%), Berg Balance Scale – balance (MD = 0.73%, CI 95% = −1.01 to 2.47, *p* = 0.41, *I*^2^ = 0%). There was no significant effect of single vs. multiple targets in: UPDRS III – motor aspects (MD = 2.05%, CI 95% = −1.96 to 6.06, *p* = 0.32, *I*^2^ = 0%) and gait (SMD = −0.05%, 95% CI = −0.28 to 0.17, *p* = 0.64, *I*^2^ = 0%). Simple univariate meta-regression analysis between treatment dosage and effect size revealed that number of sessions (estimate = −1.7, SE = 1.51, z-score = −1.18, *p* = 0.2, IC = −4.75 to 1.17) and cumulative time (estimate = −0.07, SE = 0.07, z-score = −0.99, *p* = 0.31, IC = −0.21 to 0.07) had no significant association.

**Conclusion:** There was no significant tDCS alone short-term effect on motor function, balance, gait, dyskinesias or motor fluctuations in Parkinson's disease, regardless of brain area or targets stimulated.

## Introduction

Parkinson's disease (PD) is a chronic, multisystemic, neurodegenerative disorder with various mechanisms underlying its neuropathology (1). PD is standing out as a leading cause of disability-adjusted life year (DALY) globally (increasing 148% between 1990 and 2016), the most growing neurological disorder according to the Global Burden of Disease 2016 (2), and affecting 6.1 million people (3). As an aggravating factor, the forecast predicts that this number will double in the next generation (3).

Parkinson's disease is characterized by a triad of cardinal symptoms (bradykinesia, tremor, and rigidity). Bradykinesia or slowness of movement is the most characteristic motor symptom (4), covering many motor manifestations (5). Tremor initially appears unilateral and progresses to bilateral, worsening in stressful circumstances or cognitive tasks, and can be attenuated during sleep or movement (6). Rigidity causes constant or oscillating resistance to passive joint movement and can be increased by tasks demanding attention (7).

Among the current treatments available, drug administration is the most common option. However, a significant decrease in response to a drug occurs ~5 years after initial treatment, worsening motor fluctuations, dyskinesia, dystonia, incoordination, and arthralgia (9). Neurosurgical procedures involving deep brain stimulation are another option, but this method presents high cost (9), surgical risk (8), and possibility of worsening of verbal fluency and axial motor symptoms (8, 9). Appropriate interventions present little or no adverse effects, improve functionality and well-being, and delay the progression of the disease (9). Thus, new therapeutic approaches are necessary to provide a better quality of life and to reduce the financial burden for society and health systems.

Transcranial direct current stimulation (tDCS) has gained prominence for being a non-invasive, safe, low-cost neuromodulatory modality, with minimal or no adverse effect (10, 11). Its mechanisms of action go far beyond the elementary reasoning that anodic (a-tDCS) and cathodic (c-tDCS) stimulation increases or decreases, respectively, somatic polarity, excitability, and neuronal plasticity (12). Considering the complex functioning of the brain, the neurophysiology underlying tDCS is much more heterogeneous. It can encompass the following: complex forms of plasticity, involving distinct presynaptic and postsynaptic mechanisms (long-term potentiation and depression), soma polarization, dendrites, and synaptic terminals, axonal growth, network effects (amplifications and oscillations), and functions of interneurons, endothelial cells, and glia (13). Given the pathophysiological complexity of PD and the variability of its symptoms, multiple brain regions can modulate motor recovery, and consequently, the methods of applying tDCS can be diverging.

Previous reviews investigated tDCS and associated therapies (14–19), but tDCS alone is still a challenge to determine its clinical effect on PD (15). Thus, this systematic review and meta-analysis investigated the use of tDCS on PD based on the PICOS model: population (P): adult patients with PD; intervention (I): tDCS alone in different brain areas and number of stimulated nominal targets; comparison (C): control condition, placebo or sham; outcomes (O): PD motor symptoms; types of studies (S): clinical trials randomized or not with crossover or parallel design and open-label studies.

## Methods

### Protocol and Registration

A systematic review with meta-analysis and meta-regression was performed according to the Cochrane group (20), including review mechanisms, inclusion or exclusion criteria, search and selection of articles, analysis of the methodological quality of included studies, data extraction, and meta-analysis of results. The Preferred Reporting Items for Systematic Reviews and Meta-Analyses (PRISMA) guidelines were adopted (21). The selection of studies was performed by two independent reviewers (PCAO and TABA) according to the previously structured eligibility criteria. Disagreements between reviewers were resolved by a third reviewer (DGSM). The current review protocol was registered in the International Prospective Register of Systematic Reviews – PROSPERO–(https://www.crd.york.ac.uk/prospero/) under the publicly available registry number CRD42020188010 (https://www.crd.york.ac.uk/prospero/display_record.php?ID=CRD42020188010).

### Search Strategy

The following databases were used for this review's literature survey: Cochrane Library, EMBASE, PubMed/MEDLINE, Scopus, and Web of science, and considered the literature until February 2021. The terms MeSh and operators Booleans were as follows: “Parkinson's disease” OR “Parkinson's disease” AND “transcranial direct current stimulation” OR “tDCS” OR “transcranial electrical stimulation” OR “non-invasive brain stimulation” OR “neuromodulation.” In addition, the reference lists of selected articles and literature reviews on the subject were checked to retrieve articles that were not covered by the database searches.

### Eligibility Criteria

The search was carried out for full text articles, peer-reviewed, published in scientific journals without language restriction. However, only studies in English were found. To be included, studies should (a) include adults (over 18 years of age) with a clinical diagnosis guided by the Movement Disorder Society diagnostic criteria for PD (5), all types and levels of severity or by a clinical definition; (b) apply a-tDCS or c-tDCS; (c) report motor outcome data only from individuals with PD; (d) report data on motor outcomes only from the intervention with tDCS alone (for studies involving multiple interventions); (e) provide quantitative data for at least one of the outcome measures (in the manuscript or upon request); (f) have randomized and non-randomized clinical trials with parallel, crossover, or open-label design; and (g) have a sham or control condition. Studies involving research on animals, *in vitro* or computational models, were excluded. The agreement between reviewers for the screening of studies was analyzed using the Kappa (K) statistic, and the results revealed an “excellent” agreement (*K* = 0.969; *p* < 0.0001). The percentage of agreement between the reviewers was 99.9%, and the third reviewer's tie was 0.1%.

### Study Quality Assessment

The evaluation of the internal validity and presentation of necessary statistical information of the studies was performed by two independent reviewers (PCAO and TABA), who used the classification scale of the Physiotherapy Evidence Database (PEDro) (22). The PEDro scale consists of 11 items that assess the followings: (1) eligibility criteria, (2) randomness of groups, (3) secret allocation, (4) homogeneity between groups, (5) blinding of participants, (6) blinding of therapists, (7) evaluator blinding, (8) key outcome in more than 85% of subjects, (9) intention-to-treat analysis, (10) statistical comparison between groups, and (11) precision measure and variability measures. The PEDro scale is one of the most used instruments in rehabilitation to assess the methodological quality of clinical trials (23, 24). Thus, it is a measure with sufficient validity to be used in systematic reviews of clinical trials and clinical practice guidelines (22). The classification of the PEDro score was as follows: scores from 0 to 4 = low quality; 4 to 5 = acceptable quality; 6 to 8 = good quality, and 9 to 10 = excellent quality (25).

The risk of bias was evaluated using the Cochrane risk of bias assessment (26), which assesses the followings: (a) random sequence generation, (b) allocation concealment, (c) blinding of participants and personnel, (d) blinding of outcome assessment, (e) incomplete outcome data, (f) selective reporting, and (g) other biases. Each item was classified as “low risk of bias” (“+”), “high risk of bias” (“–”) or “uncertain risk of bias” (“?”). Disagreements were resolved by a third reviewer (DGSM).

### Data Extraction

Data extraction included sample size (number of individuals involved), participant characteristics [age, gender, time since PD diagnosis, Unified Parkinson's Disease Rating Scale (UPDRS) at baseline, medication, most affected hemibody, stage Hoehn and Yahr (HY)], intervention protocol (number of sessions, location of electrodes, anodic or cathodic, intensity, density, and duration of stimulation), and outcome measures (gait, motor function, motor aspects of daily life, dyskinesia, motor fluctuations, bradykinesia, manual dexterity, upper limb function, balance, postural stability, and freezing of gait) from all included studies. Missing article data were requested by email, and those who did not respond after three attempts or did not provide data for any reason were excluded from the meta-analysis. Thus, we excluded 15 articles from the quantitative synthesis, 11 for lack of response (27–37) and 4 for not having or not providing the data (38–41).

### Quantitative Analysis

Quantitative synthesis was performed by combining individual studies into meta-analyses. We performed analyses comparing the effect of tDCS alone on motor symptoms according to the nominal stimulated target and compared the effect on single or multiple targets. To estimate the effect, we used continuous post-intervention mean and standard deviation data. We calculated the mean difference (MD) or the standardized mean difference (SMD), if the studies assessed the same outcome using different scales, confidence interval (CI) of 95% for each comparison, weighted by the inverse variance method using an effects model random or fixed-effects model, when applicable. Heterogeneity was assessed using chi-square (*p* < 0.1 = statistically significant), *I*^2^ (*I*^2^ > 75% = significant) and visual inspection of forest plots. If considerable heterogeneity was identified (chi-square *p* < 0.10; *I*^2^ > 75%), only a qualitative synthesis would be presented. Review Manager v.5.3 software (Copenhagen: Nordic Cochrane Center) was used for all data analysis, except for the meta-regression, performed in Python. The univariate meta-regression model used a sensitivity analysis to investigate possible effect moderators related to treatment characteristics (number of sessions and cumulative time). One predictor variable was analyzed at a time, and values of *p* < 0.05 were considered significant.

## Results

### Overview

**Figure 1:**
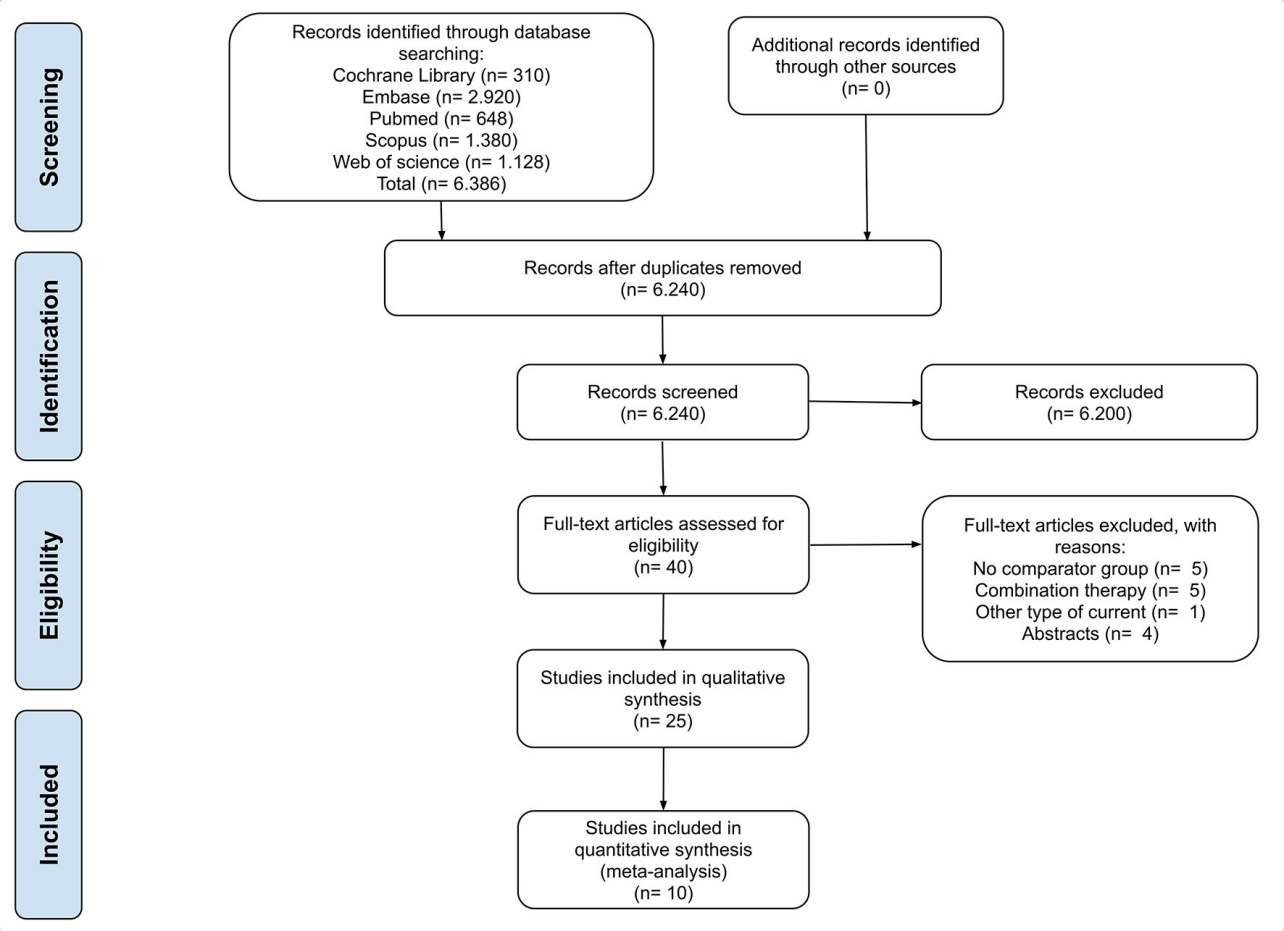
PRISMA flowchart of included studies.

This review comprises the range from 1984 to February 2021. The PRISMA flow diagram summarizes steps in the study identification procedures (Figure 1). The literature search identified 6,386 studies, and Mendeley software excluded 146 duplicates. No study was included based on verifying the reference lists of selected articles or literature reviews on the subject. Forty studies were eligible for full-text reading after evaluating titles and abstracts. The two most frequent causes of exclusion were an absence of a comparator and tDCS as a combined therapy. Another four studies were excluded after the analysis of abstracts. Studies that investigated non-motor outcomes after tDCS were also checked for the existence of motor outcomes for inclusion in the meta-analytic analysis. Finally, 25 studies involving 405 participants met our criteria and were included in the qualitative synthesis. Of those, 10 were included in the meta-analysis, covering a total of 236 participants.

### Characteristics of Included Studies

Table 1 summarizes information of included studies, which investigated the effect of tDCS alone on the motor symptoms of PD. According to this table, 20 (80%) studies were randomized (27–29, 32, 34, 36–40, 42–51), four (16%) did not mention this information (31, 33, 35, 41) and one (4%) used pseudo-randomization (30). Twenty studies (80%) had a crossover design (28–37, 40, 41, 43, 44, 46–51) and five (20%) parallel (27, 38, 39, 42, 45). One (4%) study did not contain information about blinding (39), three (12%) trials had single-blind experiments (33, 44, 50), 20 (80%) double-blind experiments (27–32, 34, 36–38, 40–43, 45–51) and one (4%) double blind in only one of the experiments (35). Regarding the comparator group, 24 studies (96%) had a sham group (27–38, 40–51) and one (4%) had a control group, which did not undergo any type of therapy (39).

**Table 1:** Characteristics of the included studies.

**References**	**Design**	**Outcome measures**	**Follow-up**	**N sessions**	**Nominal target**	**Target**	**tDCS Set-up**	**Results**
Albuquerque et al. (27)	Parallel	PGT, AMT	NO	1	(+): cerebellum & (–): buccinator muscle	Single	2 mA, 25 min, ND	Motor performance (=) in hand and arm tasks
Benninger et al. (42)	Parallel	10MWT, Hand and arm movements (bradykinesia), UPDRS, SRTT	1 and 3 months	8	(+): PMC and MC & (–): Mastoids and (+): PFC & (–): Mastoids Sham: (+) and (–) 1 cm apart over the forehead, two additional electrodes inversely over the mastoids (not connected to the stimulator)	Multi	2 mA, 20 min, 0.021 mA/cm^2^	↓ in walking time (ON and OFF) until 1 month later in the ON group improvement in bradykinesia (ON and OFF) for more than 3 months (=) for UPDRS, SRTT
Beretta et al. (28)	Cross	UPDRS, Postural control assessment, EMG, fNIRS, MMSE	NO					EMG and CoP temporal parameters: (↓) recovery time x sham
Exp 1				1	(+): M1 hemisphere contralateral to the most affected body side & (–): over the contralateral supraorbital	Single	1 mA, 20 min, ND	
Exp 2				1	(+): M1 hemisphere contralateral to the most affected body side & (–): over the contralateral supraorbital	Single	2 mA, 20 min, ND	EMG and CoP temporal parameters: (↓) onset latency with 2 mA, (↓) recovery time x sham
Bueno et al. (43)	Cross	TUG, video gait analysis	NO	1	(+): L-DLPFC & (–): R-frontal areas	Single	2 mA, 20 min, 0.057 mA/cm^2^	(=) TUG and video gait analysis
Cosentino et al. (29)	Cross	FT, upper limb bradykinesia test, UPDRS III	NO	2	(+): M1 & (–): contralateral orbitofrontal cortex; (+): contralateral orbitofrontal cortex & (–): M1	Single	2 mA, 20 min, ND	a-tDCS in most affected M1: improvement in FT, (↓) in Upper Limb Bradykinesia test time in both hands, (↓) in UPDRS III c-tDCS in less affected M1: improvement in FT, (↓) in Upper Limb Bradykinesia test time in both hands c-tDCS in most affected M1: (↑) at the time of the upper limb bradykinesia test
Criminger et al. (44)	Cross	TUG	NO	1	(+): L-DLPFC & (–): R-DLPFC	Single	2 mA, 20 min, ND	(=) TUG
da Silva et al. (45)	Parallel	Gait kinematics analysis, UPDRS III	NO	1	(+): M1 and SMA & (–): over the supraorbital area ipsilateral to the most affected side	Multi	2 mA, 15 min, ND	(↓) in gait cadence
Dagan et al. (46)	Cross	TUG, FOG-provoking test	NO	2	(+): M1 motor leg-area & (–): ND; (+): L- DLPFC and M1 & (–): ND	Single & Multi	2 mA, 20 min, ND	a-tDCS in M1 + DLPFC: (↓) in FOG-Provoking Test and TUG
Doruk et al. (38)	Parallel	UPDRS III, sRT, 4-CRT, PPT, FT, WT, BU, SP	1 month	10	(+): L-DLPFC & (–): R-frontal areas; (+): R-DLPFC & (–): L-frontal areas	Single	2 mA, 20 min, ND	(=) motor function
Ferrucci et al. (47)	Cross	UPDRS III/IV	1 and 4 weeks	5	(+): M1 bilaterally & (–): R-deltoid muscle; (+): cerebellum & (–): R-shoulder	Single	2 mA, 20 min, ND	a-tDCS in M1 and cerebellum improved levodopa-induced dyskinesias
Fregni et al. (30)	Cross	UPDRS, sRT, PPT	NO	1	(+): M1 dominant hemisphere & (−): contralateral orbitofrontal cortex (+): contralateral orbitofrontal cortex & (–): M1 dominant hemisphere (+): DLPFC & (–): orbitofrontal cortex	Single	1 mA, 20 min, ND	a-tDCS in M1: improvement in UPDRS and sRT, (=) for PPT a-tDCS in DLPFC: significant main effect for UPDRS and sRT, (=) for PPT c-tDCS in M1: (=) for UPDRS, sRT and PPT
Kaski et al. (48)	Cross	6MWT, gait velocity, stride length, TUG, pull test	NO	1	(+): M1 (leg areas, 10–20% anterior to Cz) & (–): inion	Single	2 mA, 15 min, ND	(=) gait speed, stride length, TUG, 6MWT and pull test
Lattari et al. (49)	Cross	BBS, DGI, TUG	NO	1	(+): L-DLPFC & (–): R-frontal areas	Single	2 mA, 20 min, ND	a-tDCS improves balance and functional mobility x sham-tDCS
Lawrence et al. (39)	Parallel	UPDRS II	week 12	4	(+): L-DLPFC & (–): above the left eye	Single	1.5 mA, 20 min, ND	Isolated tDCS did not generate significant improvement in any motor test
Lu et al. (40)	Cross	UPDRS III, gait initiation on the force platform	NO	1	(+): SMA & (–): Fp	Single	1 mA, 10 min, 0.123 mA/cm^2^	a-tDCS did not improve self-start gait in PD and FOG
Manenti et al. (31)	Cross	TUG	NO	1	(+): L-DLPFC & (–): R-frontal areas; (+): R-DLPFC & (–): L-frontal areas	Single	2 mA, 7 min, 0.057 mA/cm^2^	(↓) Selective on TUG reaction times after a-tDCS on R-DLPFC and (=) L-DLPFC
Mishra and Thrasher (32)	Cross	GAITRite (velocity), phoneme verbal fluency task	15 and 30 min	1	(+): L-DLPFC & (–): R-frontal areas	Single	2 mA, 30 min, ND	a-tDCS x sham in the dual task: participants walked faster and generated a (↑) number of words/min, at 15 and 30 min after stimulation The cost of dual task associated with gait speed was significantly (↓) 15 min after Single task: (=) for gait and cognitive performance
Putzolu et al. (33)	Cross	GAITRite	NO	1	(+): L-DLPFC & (–): R-frontal areas	Single	1.5 mA, 20 min, ND	Improvement in gait performance during cognitive dual task in the FOG group
Putzolu et al. (34)	Cross	GAITRite	NO	1	(+): L-DLPFC (–): orbitofrontal cortex	Single	1.5 mA, 20 min, ND	Improved stride length, stride speed and double support time
Salimpour et al. (35)			NO					(↓) on signal-dependent noise in the most affected arm (↑) on patients' willingness to assign strength to the most affected arm and improvement of motor symptoms
Exp 1	Cross	Isometric task, UPDRS III	NO	1	(+): L-M1 & (–): R-M1	Single	1 mA, 25 min, 0.04 mA/cm^2^	-
Exp 2	Cross	Isometric task, UPDRS III	NO	1	(+): R-M1 & (–): L-M1	Single	2 mA, 25 min, 0.08 mA/cm^2^	(↓) in the subjective cost of force (↑) in the willingness to assign force to the affected side (↓) in noise laterality
Exp 3	Cross	Isometric task, UPDRS III	NO	2	(+): M1 contralateral to the affected side & (–): M1 contralateral to the affected side	Single	2 mA, ND, 0.08 mA/cm^2^	(↑) in the willingness to give strength to the affected side (↓) in the laterality index (↓) at UPDRS
Exp 4	Cross	Isometric task, UPDRS III, PDQ-39	NO	5	(+): M1 ipsilateral & (–): M1 contralateral to the affected side	Single	2 mA, ND, 0.08 mA/cm^2^	c-tDCS x sham: further improvements in the laterality index (↓) higher in the subjective cost of strength in the affected arm change in one-hand noise significant effect on UPDRS improvement in PDQ-39
Schoellmann et al. (36)	Cross	UPDRS III, EMG, EEG	30 min	1	(+): M1 & (–): R-frontal areas	Single	1 mA, 20 min, <0.1 mA/cm^2^	Clinical motor improvement of the UPDRS III subtotal (items 22–25) of the MSD lasting at least 30 min
Swank et al. (50)	Cross	TUG, PDQ-39, UPDRS	NO	1	(+): L-DLPFC & (–): R-DLPFC	Single	2 mA, 20 min, ND	(=) TUG or PDQ-39
Valentino et al. (37)	Cross	UPDRS III and total, SWS, FOG-Q, GFQ	2 days, 2 and 4 weeks	5	(+): M1 (leg area that starts walking) & (–): contralateral orbitofrontal cortex	Single	2 mA, 20 min, ND	Improved gait (↓) on the number and duration of FOG episodes (↓) in total UPDRS and III
Verheyden et al. (41)	Cross	STS, FR, SS180, TUG, 10MWT	NO	1	(+): M1 of the dominant hemisphere & (–): contralateral orbitofrontal cortex	Single	1 mA, 15 min, ND	(↓) of speed at 10MWT no immediate effects and, in fact, a possible decline in motor performance
Workman et al. (51)	Cross	25FWT, TUG, 6MWT, BBS, Posturography	NO	1	Unilateral (+): Hemisphere cerebellar more affected & (–): Contralateral upper arm Bilateral (+): Hemisphere cerebellar more affected & (–): hemisphere cerebellar contralateral	Single	2 mA, 20 min, 0.06 mA/cm^2^ 4 mA, 20 min, 0.11 mA/cm^2^	4 mA bilateral: (↑) on the BBS

*N, number; PGT, precision grip task; AMT, arm movement task; mA, milliamps; min, minutes; ND, not described; 10MWT, 10 m; UPDRS, Unified Parkinson's Disease Rating Scale; walk test; SRTT, serial reaction time task; PMC, premotor cortex; MC, motor cortex; PFC, prefrontal cortex; EMG, electromyography; fNIRS, functional near-infrared spectroscopy; MMSE, mini mental state examination; CoP, center of pressure; Exp, experiment; M1,primary motor cortex; TUG, timed up and go; L, left; DLPFC, dorsolateral prefrontal cortex; R, right; FT, finger tapping; a-tDCS, anodal transcranial direct current stimulation; c-tDCS, cathodal transcranial direct current stimulation; SMA, supplementary motor area; FOG, freezing of gait; sRT, simple reaction time; 4-CRT,4-choice reaction time; PPT, Purdue Pegboard test; WT, walking time; BU, buttoning-up; SP, supination–pronation; 6MWT, six-min walk test; BBS, Berg Balance Scale; DGI, Dynamic Gait Index; Fp, frontal polar; PD, Parkinson's disease; GAITRite, gait assessment system; PDQ-39, Parkinson's Disease Questionnaire 39 Items; EEG, electroencephalography; MSD, superior right member; SWS, stand–walk–sit; FOG-Q, Freezing of Gait Questionnaire; GFQ, Gait and Fall Questionnaire; STS, sit-to-stand; FR, functional reach; SS180, standing-start 180 degrees turning; 25FWT,25-foot walk test; Cross, crossover design; parallel, parallel design; Single, single target; Multi, multiple targets; (↑), increase; (↓), decrease; (=), equal*.

### Characteristics of Participants

In total, 25 studies included 405 individuals with PD, and the mean sample size was 17.64 ± 7.40 (ranging from 7 to 26 participants), aged between 58 and 74 years. HY obtained a minimum score of 1.3 and a maximum of 2.8, indicating early to almost moderate stages of PD. The UPDRS II achieved a minimum score of 1.1 and a maximum of 11.6, a UPDRS III minimum of 13 and a maximum of 39.7, and a minimum of 16 and a maximum of 74.2 on UPDRS's total score. The duration of PD had a minimum of 4.3 and a maximum of 12.3 years whereas the dose of the medication had a minimum of 292.8 mg and a maximum of 1287.7 mg. Twenty-two (88%) studies performed the experiment in the ON state of the medication (27–35, 37–39, 41, 43–51), two (8%) in the OFF state (36, 40) and one (4%) in both states (42). Details of the participants of each study are shown in Table 2.

**Table 2:** Characteristics of participants.

**References**	**Sample (W/M)**	**Age (years)**	**Hoehn and Yahr**	**Duration of disease (years)**	**UPDRS at baseline**	**Medication (mg)**	**Most affected hemibody (Right, Left, Bilateral)**	**ON/OFF phase**
Albuquerque et al. (27)	22 (10 W/12 M)	71.3 ± 8.6	Active: 2.3 ± 0.65 Sham: 2.0 ± 0,63	ND	Active: 24.7 ± 5.7 Sham: 28.4 ± 12.1 (ND)	Active: 584.8 ± 516.2 Sham: 468.5 ± 193.7	20R/2L	ON
Benninger et al. (42)	25 (9 W/16 M)	63.9 ± 8.7	Active: 2.5 ± 0.1 Sham: 2.4 ± 0.2	Active: 10.6 ± 7.1 Sham: 9.1 ± 3.3	Active: 42.5 ± 10.8 Sham: 39.5 ± 12.8 (total) Active: 22.2 ± 8.7 Sham: 17.5 ± 8 (III)	Active: 1024.3 ± 541.5 mg Sham: 1287.7 ± 808.8 mg	ND	ON/OFF
Beretta et al. (28)	24 (10 W/14 M)	68.91 ± 8.47	ND	4.84 ± 3.11	36.00 ± 14.32 (III)	545.01 ± 288.59 mg	ND	ON
Bueno et al. (43)	20 (8 W/12 M)	64.45 ± 8.98	2.25 ± 0.63	7.80 ± 5.32	11.60 ± 4.00 (II) 22.35 ± 6.77 (III) 33.95 ± 9.44 (total)	ND	ND	ON
Cosentino et al. (29)	14 (6 W/8 M)	58 ± 12.1	ND	ND	ND	386.2 ± 233.5 mg	11R/3L	ON
Criminger et al. (44)	16 (4 W/12 M)	68.13 ± 9.76	2 ± ND	8.69 ± 9.76	40.31 ± 18.27 (total) 23.44 ± 9.73 (III)	ND	ND	ON
da Silva et al. (45)	17 (7 W/10 M)	Active: 66 ± 5 Sham: 66 ± 10	2.35 ± 0.29	Active: 6 ± 6 Sham: 5 ± 1	ND	ND	ND	ON
Dagan et al. (46)	20 (3 W/17 M)	68.8 ± 6.8	2.5 ± 0.6	9.0 ± 5.7	74.2 ± 23.3 (total) 39.7 ± 14.6 (III)	554.7 ± 401.1 mg	ND	ON
Doruk et al. (38)	18 (6 W/12 M)	61 ± 8	ND	ND	ND	ND	ND	ON
Ferrucci et al. (47)	9 (4 W/5 M)	74.33 ± 7.98	2.5 ± 0.35	10.77 ± 2.1	Active Cerebellar: 13 ± 4.9 Active M1: 13 ± 4.8 Sham: 13.3 ± 4.8 (III)	ND	ND	ON
Fregni et al. (30)	17 (6 W/11 M)	62.3 ± 1.6	2.4 ± 0.2	12.3 ± 1.6	37.4 ± 3.9 (III)	615.0 ± 63.1 mg	9R/8L	ON
Kaski et al. (48)	8 (ND)	ND	ND	ND	25.8 ± 5.74 (total)	ND	ND	ON
Lattari et al. (49)	17 (4 W/13 M)	69.18 ± 9.98	2.35 ± 1.06	7.06 ± 2.70	18.0 ± 8.96 (III)	748.29 ± 343.80 mg	ND	ON
Lawrence et al. (39)	tDCS: 7 (5 W/2 M) control: 7 (4 W/3 M)	tDCS: 72 ± 6.45 control: 72.29 ± 6.21	ND	tDCS: 5.50 ± 5.66 control: 5.36 ± 4.14	tDCS: 1.27 ± 0.56 (II) control: 1.18 ± 0.69 (II)	tDCS: 573.29 ± 586.25 control: 292.88 ± 274.51	ND	ON
Lu et al. (40)	10 (3 W/7 M)	66.3 ± 9.9	2.7 ± 0.4	7.7 ± 4.0	39.2 ± 17.2 (III)	761.0 ± 362.2 mg	ND	OFF
Manenti et al. (31)	10 (4 W/6 M)	67.1 ± 7.2	1.3 ± 1.1	8.1 ± 3.5	13.3 ± 5.7 (III)	749.2 ± 445.5 mg	2R/8L	ON
Mishra and Thrasher (32)	20 (6 W/14 M)	67.8 ± 8.3	1.9 ± 0.9	4.8 ± 3.8	ND	ND	ND	ON
Putzolu et al. (33)	20: FOG+ (4 W/6 M) FOG- (5 W/5 M)	FOG+: 70.1 ± 3.84 FOG-: 72.8 ± 6.87	ND	FOG+: 9.3 ± 5.5 FOG-: 7.2 ± 5.2	FOG+: 20.1 ± 8.4 (III) FOG-: 22.9 ± 8.1 (III)	ND	ND	ON
Putzolu et al. (34)	21: FOG+ (4 W/6 M) FOG- (4 W/7 M)	FOG+: 69.20 ± 5.20 FOG-: 70.36 ± 6.23	FOG+: 2.05 ± 0.44 FOG-: 1.77 ± 0.52	FOG+: 8.00 ± 5.50 FOG-: 5.82 ± 5.29	FOG+: 39.30 ± 11.39 (total) FOG-: 36.27 ± 16.58 (total) FOG+: 18.60 ± 6.38 (III) FOG-: 20.45 ± 8.15 (III)	ND	ND	ON
Salimpour et al. (35)								ON
Exp 1	10 (4 W/6 M)	59.6 ± 6.68	1.75 ± 0.54	6.9 ± 4.6	15.7 ± 4.8 (III)	515 ± 274.92	10R/0L	
Exp 2	10 (2 W/8 M)	61.6 ± 10.76	1.75 ± 0.63	8.5 ± 5.8	18.6 ± 6.09 (III)	655 ± 434.90	10R/0L	
Exp 3	10 (4 W/6 M)	60.5 ± 9.16	1.85 ± 0.47	8.3 ± 4.13	24.6 ± 11.21 (III)	740 ± 500.99	8R/1L/1B	
Exp 4	8 (3W/5M)	59.37 ± 9.00	1.5 ± 0.46	6.87 ± 4.96	17.62 ± 4.47 (III)	712.5 ± 470.37	6R/2L	
Schoellmann et al. (36)	10 (4 W/6 M)	64.3 ± 11.4	ND	8.6 ± 4.1	ND	749.15 ± 423.99 mg	7R/3L	OFF
Swank et al. (50)	10 (2W/8M)	68.7 ± 10.2	2 ± ND	7.9 ± 7.1	37.0 ± 12.9 (total) 24.30 ± ND (III)	ND	ND	ON
Valentino et al. (37)	10 (5 W/5 M)	72.3 ± 3.6	2.8 ± 0.5	11 ± 4.9	32 ± 10.3 (III)	ND	4R/6L	ON
Verheyden et al. (41)	20 (ND)	71 ± 7	ND	9 ± 4	16 ± 5 (total)	ND	ND	ON
Workman et al. (51)	7 (2W/5M)	72.4 ± 6.4	1.9 ± 0.4	4.3 ± 2.5	32.6 ± 14.2 (III)	889.8 ± 497.7 mg	1R/6L	ON

*W, women; M, men; UPDRS, Unified Parkinson's Disease Rating Scale; mg, milligrams; ND, not described; R, right; L, left; FOG, freezing of gait; Exp, experiment*.

### tDCS Protocols

**Figure 2:**
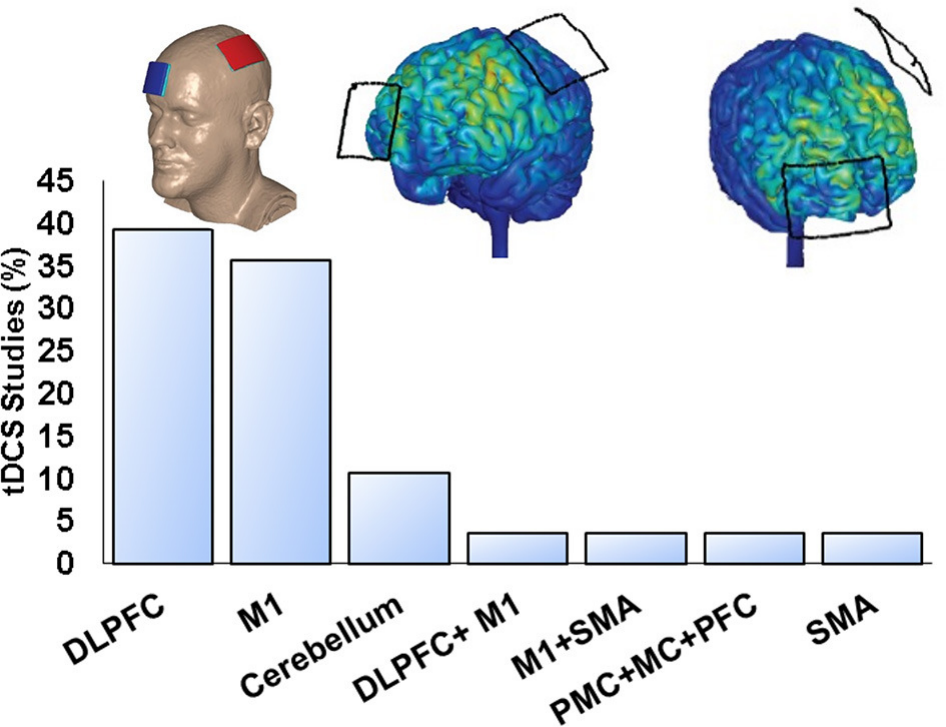
Quantity of tDCS studies on PD using single nominal targets, and example of M1 anodal electrode on C3.

Three (12%) studies stimulated multiple targets, and 22 studies (88%) stimulated single nominal target with dorsolateral prefrontal cortex (DLPFC) and primary motor cortex (M1) as the most common montages (Figure 2). In addition, most studies performed single tDCS session (66.7%), and others used 2 (11.1%), 4 (3.7%), 5 (11.1%), 8 (3.7%), and 10 sessions (3.7%). Twenty-two trials (88%) applied anodal tDCS (27, 28, 31–34, 36–51), whereas three (12%) performed anodal tDCS and cathodal tDCS (29, 30, 35). The minimum current intensity was 1 mA, the maximum was 4 mA, and the mean duration time of stimulation per session was 19.28 ± 4.47 min (minimum of 7 min and maximum of 30 min). Finally, 18 (72%) studies performed pre- and postintervention assessments (27–31, 33–35, 40, 41, 43–46, 48–51), whereas seven (28%) also performed follow-up evaluations (range from 15 min to 3 months) (32, 36–39, 42, 47).

### Motor Outcome Result Measures

Gait was analyzed in 17 (68%) of those studies (31–34, 37, 38, 40–46, 48–51) and which was evaluated by the timed up and go (TUG) test, 10-m walk test, video analysis and pressure platform, six-min walk test, stand–walk–sit test, 25-foot walk test, and Dynamic Gait Index. Thirteen studies (52%) also investigated the effect of tDCS alone on UPDRS scores (28–30, 35–40, 42, 45, 47, 50), on bradykinesia, manual dexterity, and upper limb function (27, 29, 30, 35, 38, 42). Balance or postural stability was analyzed in five (20%) studies (28, 41, 48, 49, 51) and freezing of gait (FOG) in other three (12%) studies (37, 40, 47).

### Quality of Included Studies

Internal validity and necessary statistical information were evaluated using the PEDro scale and obtained a mean score of 8.28 ± 1.24, which reveals a good methodological quality of the studies (25). Details of the scores for each study are shown in Table 3.

**Table 3:** PEDro scale.

	**Total**	**Items**										
		**1**	**2**	**3**	**4**	**5**	**6**	**7**	**8**	**9**	**10**	**11**
Albuquerque et al. (27)	8	1	0	0	1	0	1	1	1	1	1	1
Benninger et al. (42)	9	1	1	0	1	1	0	1	1	1	1	1
Beretta et al. (28)	9	1	1	0	1	1	0	1	1	1	1	1
Bueno et al. (43)	10	1	1	1	1	1	0	1	1	1	1	1
Cosentino et al. (29)	9	1	1	0	1	1	0	1	1	1	1	1
Criminger et al. (44)	8	1	1	0	1	1	0	0	1	1	1	1
da Silva et al. (45)	10	1	1	1	1	1	0	1	1	1	1	1
Dagan et al. (46)	9	1	1	0	1	1	0	1	1	1	1	1
Doruk et al. (38)	9	1	1	0	1	1	0	1	1	1	1	1
Ferrucci et al. (47)	9	1	1	0	1	1	0	1	1	1	1	1
Fregni et al. (30)	8	1	0	0	1	1	0	1	1	1	1	1
Kaski et al. (48)	8	1	1	0	0	1	0	1	1	1	1	1
Lattari et al. (49)	9	1	1	0	1	1	0	1	1	1	1	1
Lawrence et al. (39)	7	1	1	0	1	0	0	0	1	1	1	1
Lu et al. (40)	9	1	1	0	1	1	0	1	1	1	1	1
Manenti et al. (31)	8	1	0	0	1	1	0	1	1	1	1	1
Mishra and Thrasher (32)	8	0	1	0	1	1	0	1	1	1	1	1
Putzolu et al. (33)	6	0	1	0	1	1	0	0	0	1	1	1
Putzolu et al. (34)	9	1	1	0	1	1	0	1	1	1	1	1
Salimpour et al. (35)	4	0	0	0	0	1	0	0	0	1	1	1
Schoellmann et al. (36)	8	1	1	0	1	1	1	0	0	1	1	1
Swank et al. (50)	8	1	1	0	1	1	0	0	1	1	1	1
Valentino et al. (37)	9	1	1	0	1	1	0	1	1	1	1	1
Verheyden et al. (41)	8	1	0	0	1	1	0	1	1	1	1	1
Workman et al. (51)	8	0	1	0	1	1	0	1	1	1	1	1

*Items: (1) eligibility criteria, (2) group randomness, (3) secret allocation, (4) homogeneity between groups, (5) blinding of participants, (6) blinding of therapists, (7) blinding of evaluators, (8) key result in more than 85% of individuals, (9) analysis of the intention to treat, (10) statistical comparison between groups, and (11) precision measure and variability measures*.

**Figure 3:**
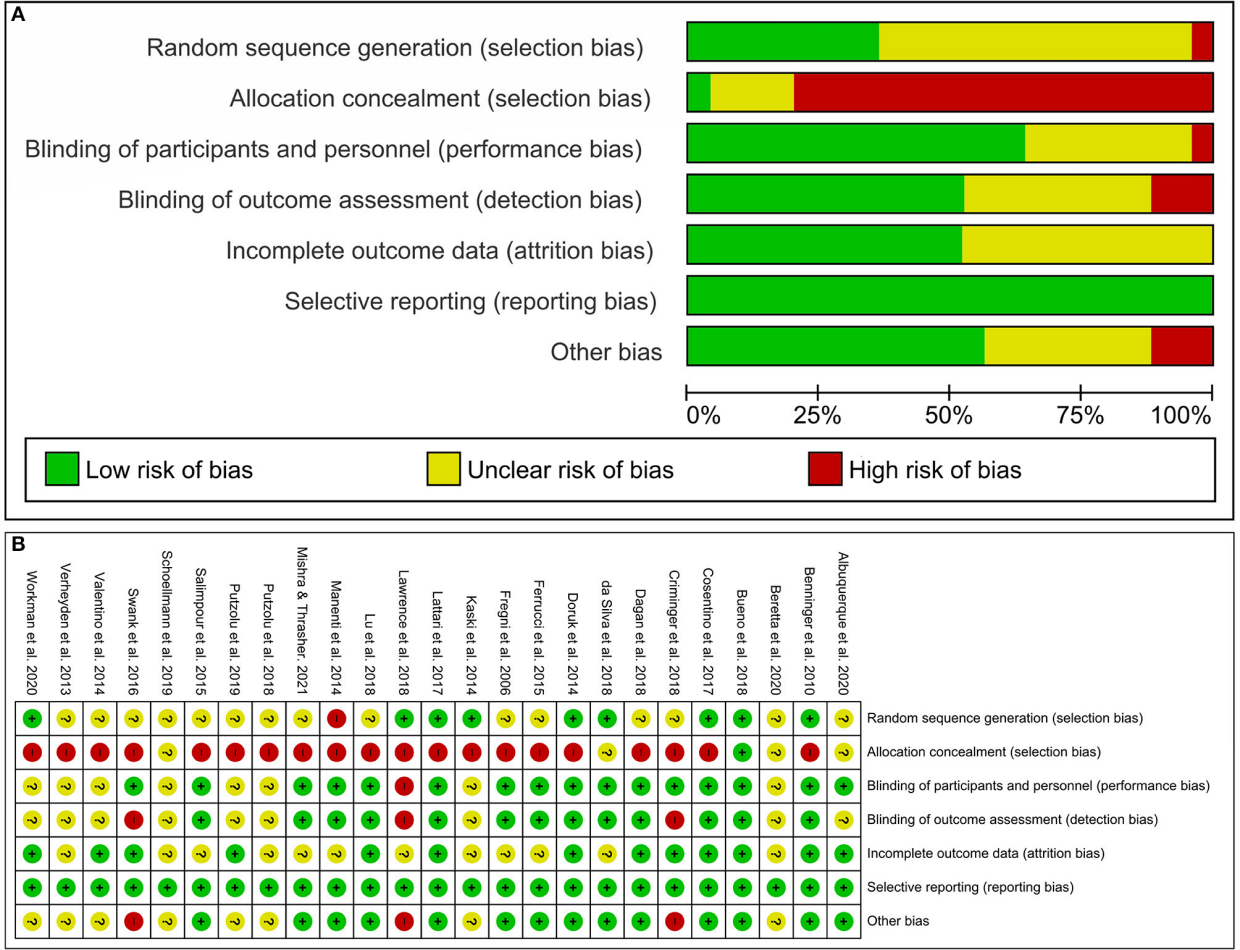
Risk of bias graph **(A)** and risk of bias summary **(B)**.

The results of risk of bias indicate a low or unclear risk for most studies except for allocation concealment that was considered high. Details of risk of bias of each study are shown in Figure 3.

### Meta-Analysis Results

#### Single Nominal Targets on Motor Symptoms

##### UPDRS III–Motor Aspects

**Figure 4:**
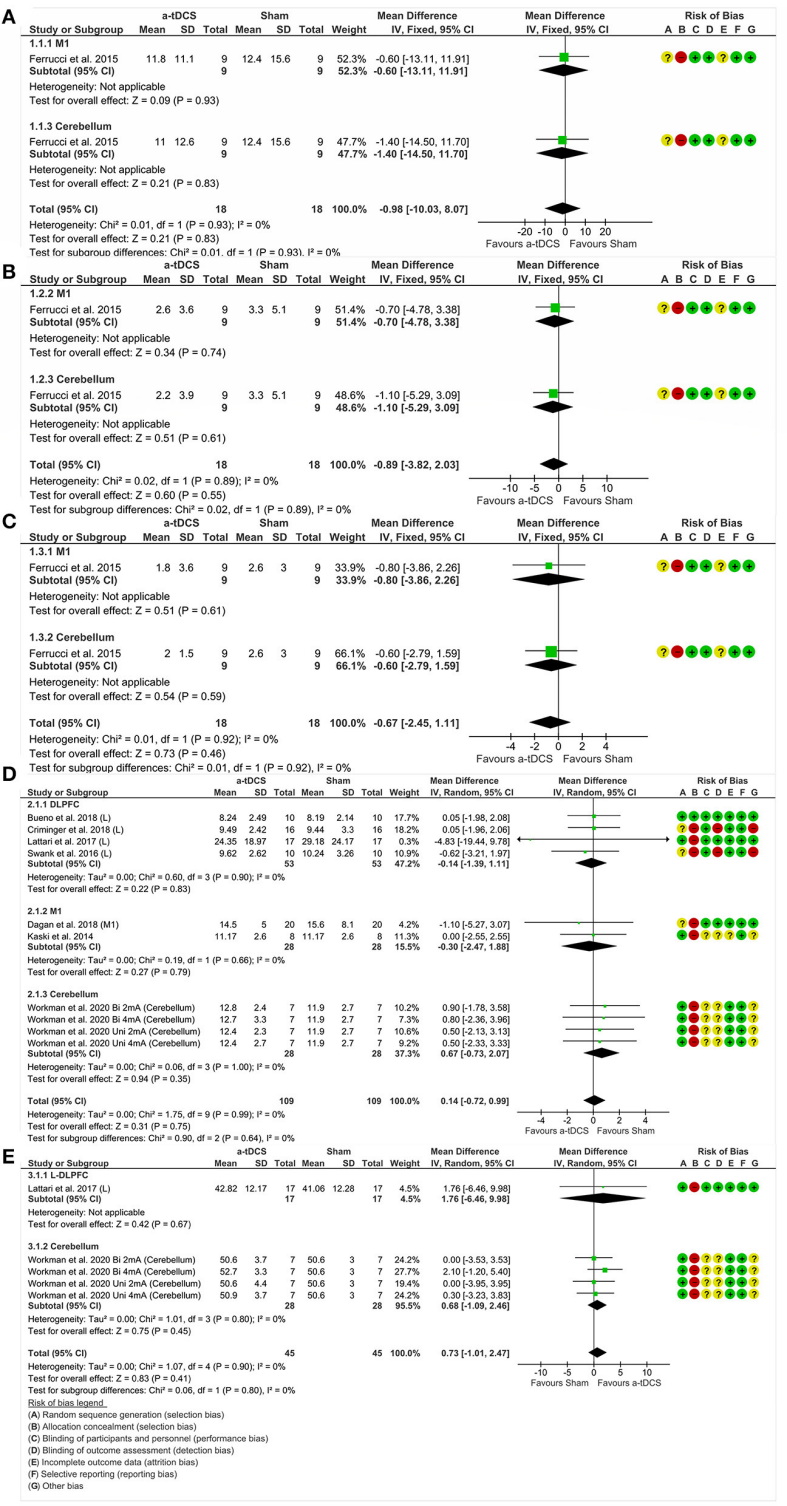
Forest plot showing mean difference from the comparison between single targets in motor function—UPDRS III **(A)** and dyskinesias—UPDRS IV **(B)** and motor fluctuations—UPDRS IV **(C)** and the gait—TUG **(D)** and balance—BBS **(E)**. Risk of bias was deemed as “low risk of bias” (“+”), “high risk of bias” (“–”), or “unclear risk of bias” (“?”).

This analysis included one study (47) and two experiments divided by nominal target, namely M1 and cerebellum. A total of nine participants were involved and randomly assigned to one stimulation protocol at once. There was no significant effect of tDCS on motor aspects measured by the UPDRS III. Analyzing the combined effect of these areas (MD = −0.98%, 95% CI = −10.03 to 8.07, *p* = 0.83, *I*^2^ = 0%, without significant heterogeneity and fixed-effects model), there was no significant effect about isolated areas (Figure 4A).

##### UPDRS IV—Dyskinesias

One study (47) enrolled 09 participants and conducted two experiments in which nominal targets, M1 and cerebellum, were stimulated. There was no significant effect between tDCS and dyskinesias assessed by the UPDRS IV. Furthermore, analyzing the combined effect of these areas (MD = −0.89%, CI 95% = −3.82 to 2.03, *p* = 0.55, *I*^2^ = 0%, without significant heterogeneity and fixed-effects model), there was also no significant effect in the analysis of isolated areas (Figure 4B).

##### UPDRS IV—Motor Fluctuations

Two experiments divided by nominal targets included M1 (47) and cerebellum (47). A total of 09 participants were involved and randomly assigned to M1, cerebellum or sham stimulation. There was no significant effect of tDCS in relation to motor fluctuations, measured by the UPDRS IV. Analyzing the combined effect of these areas (MD = −0.67%, CI 95% = −2.45 to 1.11, *p* = 0.46, *I*^2^ = 0%, without significant heterogeneity and fixed-effects model), there was also no significant effect in the analysis of isolated areas (Figure 4C).

##### TUG—Gait

We analyzed 98 participants distributed in seven studies, grouped by areas of stimulation, namely DLPFC (43, 44, 49, 50), M1 (46, 48) and cerebellum (51). There was no significant effect of tDCS in relation to gait, measured by TUG. Analyzing the combined effect of these areas (MD = 0.14%, CI 95% = −0.72 to 0.99, *p* = 0.75, *I*^2^ = 0%, without significant heterogeneity and random effects model), there was also no significant effect in the analysis of isolated areas (Figure 4D).

##### Berg Balance Scale—Balance

We compared 24 participants and protocol stimulations distributed in two studies, divided according to the areas of DLPFC (49) and cerebellum (51). There was no significant effect of tDCS related to balance, measured by the BBS. Analyzing the combined effect of these areas (MD = 0.73%, CI 95% = −1.01 to 2.47, *p* = 0.41, *I*^2^ = 0%, without significant heterogeneity and random effects model), there was no significant effect in the analysis of isolated areas (Figure 4E).

#### Single and Multiple Nominal Targets on Motor Symptoms

##### UPDRS III—Motor Aspects

**Figure 5:**
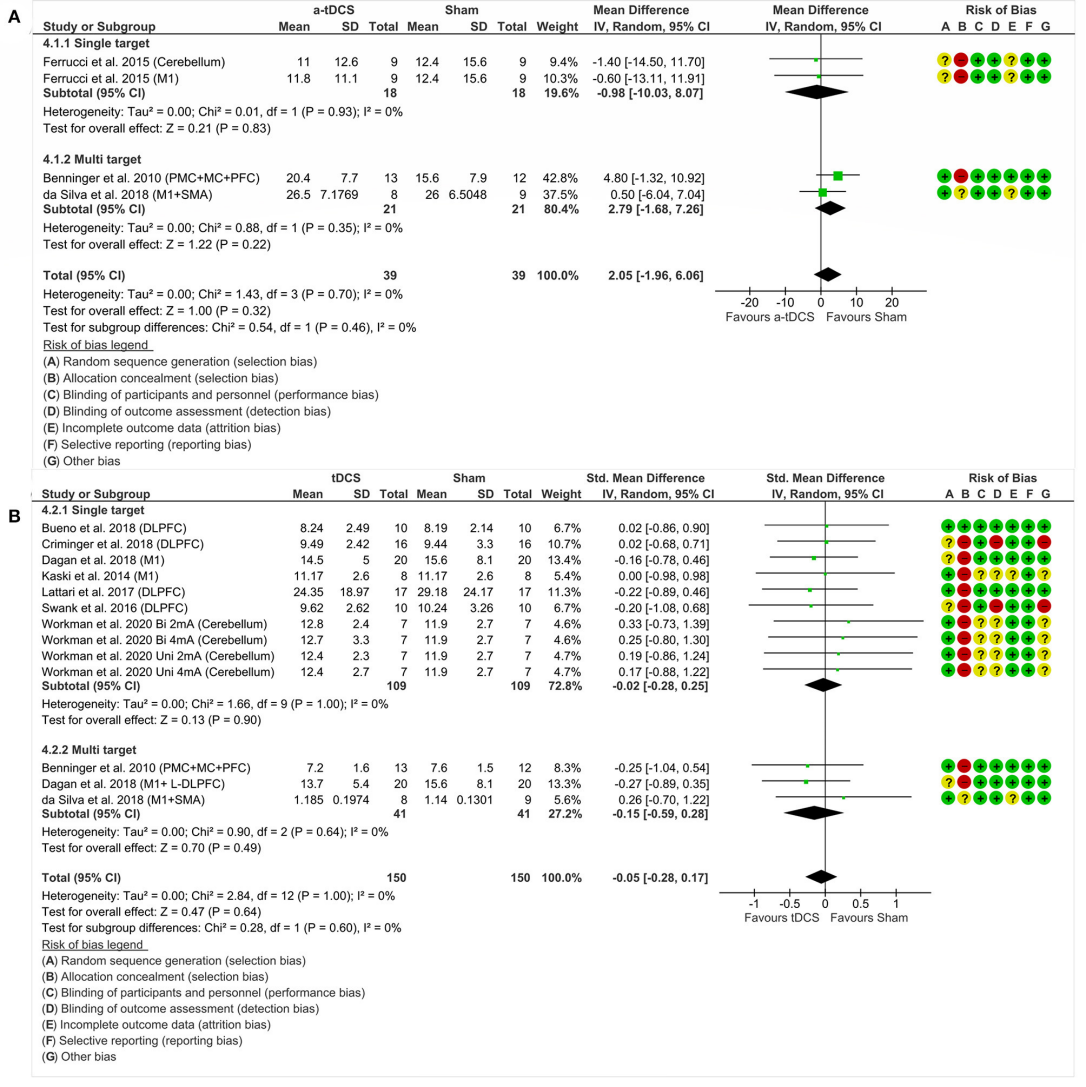
Forest plot showing mean difference from the comparison between single targets vs. multitarget in motor aspects—UPDRS III **(A)** and standardized mean difference in the gait **(B)**. Risk of bias was deemed as “low risk of bias” (“+”), “high risk of bias” (“–”), or “unclear risk of bias” (“?”).

We analyzed three studies and four experiments, with 51 participants, grouped according to the number of stimulation areas: single target (47) and multiple targets (42, 45). There was no significant effect of tDCS in relation to the motor aspects assessed by the UPDRS III. Analyzing the combined effect of these areas (MD = 2.05%, CI 95% = −1.96 to 6.06, *p* = 0.32, *I*^2^ = 0%, no heterogeneity and random effects model), there was also no significant effect in the analysis of isolated areas (Figure 5A).

##### Gait

In this analysis, we included 10 studies, with 98 participants, grouped by the amount of stimulation areas: single target (43, 44, 46, 48–51) and multiple targets (42, 45, 46). The investigation did not show a significant effect of tDCS, regardless of the number of nominal targets stimulated, in relation to gait. Analyzing the combined effect of these areas (SMD = −0.05%, 95% CI = −0.28 to 0.17, *p* = 0.64, *I*^2^ = 0%, without significant heterogeneity and random effects model), there was also no significant effect in the analysis of isolated areas (Figure 5B).

### Meta-Regression

Simple univariate meta-regression analysis was performed by a blinded investigator (ACRN) using Python “Pymare” library to investigate the association between effect size and treatment dosage considered as the number of sessions and cumulative time. Analysis revealed that the number of sessions was not significantly associated with effect size (estimate = −1.7, SE = 1.51, z-score = −1.18, *p* = 0.2, CI = −4.75 to 1.17). The analysis also revealed that cumulative time was also not significantly associated with effect size (estimate = −0.07, SE = 0.07, z-score = −0.99, *p* = 0.31, CI = −0.21 to 0.07).

## Discussion

This systematic review with meta-analysis and meta-regression includes 25 studies with 405 participants and investigated the effect of tDCS on the motor symptoms of PD. Our results demonstrated that there was no significant effect of tDCS on short-term motor symptoms of PD, regardless of brain area, number of stimulated nominal targets, or treatment dosage. The regions most covered by the included studies were DLPFC and M1.

The DLPFC is a brain region commonly studied in tDCS research to observe its effect on non-motor symptoms of PD, but it has also been widely investigated in motor symptoms. The justification includes several explanations: (a) the non-motor symptoms influence the motor symptoms because cognitive functions are needed to perform motor tasks and are partly modulated by the DLPFC (31). An example of this relationship is the execution of the gait, where the individual needs the ability to perform a dual task (43); (b) DLPFC appears to interfere with balance, through the attribution of the prefrontal cortex to spatial orientation (52) in addition to its activation during gait in several challenging conditions (53, 54). Thus, the hypothesis suggests that modulating DLPFC can improve visuospatial processing that improves the balance of individuals with PD (49). However, the literature shows divergent results related to the stimulation of this area to improve motor functions. Previous research (30, 38, 39, 43, 44, 50) found no significant effect of tDCS on DLPFC for motor function, simple reaction time, aspects of isolated and dual task gait, quality of life, or motor aspects of daily life. In contrast, other studies (31, 33, 34, 49) found a beneficial effect for walking alone and with dual task, FOG, functional mobility, or balance. Finally, responses in DLPFC can activate distinct networks of motor areas, such as M1, supplementary motor area, and premotor area, which exert direct control over motor aspects (55, 56). However, the possibility of cortical functioning through a matrix cannot be excluded, as in pain processing (12, 57).

In turn, the M1 area is also a widely investigated target for treatment of motor symptoms of PD, due to its primordial role in motor control and learning (58). In summary, the disturbance in the functioning of the basal ganglia causes cortical dysfunction and promotes the motor symptoms of PD. Thus, the hypothesis is that the modulation of cortical areas can drive changes in the cortical–subcortical pathway, positively influencing the basal ganglia, to correct such dysfunction and reduce symptoms (30). However, the literature about tDCS in M1 shows divergences. According to previous studies (29, 30, 35–37, 41, 47), tDCS in M1 showed a significant effect on hand motor performance, dyskinesia, gait, FOG, motor function, and simple reaction time, respectively. However, other studies (46, 48) did not obtain a significant effect on gait and balance.

It is important to note that despite the inaccuracies in the clinical effect, there is evidence that tDCS stimulates both the target area and beyond (12). Neurophysiological mechanisms may include changes in neuronal excitability, plasticity, neuronal oscillations, and connectivity (12). Numerous studies using electroencephalography (59–62), functional magnetic resonance (57, 63–65), combination of transcranial magnetic stimulation with electroencephalography (66, 67), and functional near-infrared spectroscopy (68) have shown brain changes after tDCS in M1 with modulation of this neural network.

According to the meta-analysis, it is still not possible to determine the number of nominal targets to be stimulated in tDCS protocols to reduce motor symptoms in PD. Considering pathophysiological mechanisms, chronic evolution, multisystem repercussions, and varied symptoms, it is necessary to note the importance of functional rehabilitation combined with additional approaches. The potential of tDCS at disease onset is also relevant as most motor treatment is provided in the early phase (during the 1st week and month) (69, 70). Here, we provide some evidences that tDCS can improve motor function in early-stage patients to some extent. In previous studies, early stimulation of tDCS reduced cadence (45), upper limb bradykinesia (29), FOG (46), and improved levodopa-induced dyskinesia (47).

There is little evidence regarding the mechanisms of action of tDCS in the pathophysiology of PD. However, our hypothesis is that multiple sessions of tDCS associated with rehabilitation training can activate brain regions by the task-related activity and, therefore, make them more sensitive to modulation by tDCS (13). Different hypotheses can explain our results: a) few studies involving PD, neuromodulation, and motor symptoms aimed to assess the isolated effect of tDCS. Furthermore, studies that proposed to investigate such aspects, an even smaller number presented essential numerical data for a meta-analytic evaluation; b) our meta-regression showed that the number and cumulative time of sessions were not associated with tDCS effect size, which may suggest insufficient corticospinal changes to increase motor performance and such insufficiency may be associated with other factors, including long interval of hours between applications and longer application time, which can inhibit overstimulation through neuronal counter-regulation, among others (71); (c) the sample size of the included trials may have been limited to provide an adequate effect size; and d) there is a lack of evidence on the non-motor aspects of PD, which may influence the effectiveness of tDCS on motor outcomes. Thus, it is likely that cognitive processing is supported by several brain regions and neural networks, which makes it challenging to identify specific nominal targets to stimulate. Furthermore, our results cannot be applicable to individuals in the OFF state of medication, as most studies (88%) performed their research only in the ON state.

This systematic review with meta-analysis and meta-regression aimed to fill the gaps in the literature related to the effect of tDCS on the motor symptoms of individuals with PD. Based on the evidence from previous meta-analyses, our study (a) provided a direct comparison between the effect sizes of studies that used motor and non-motor cortical targets, (b) compared the effects of single montages target vs. multitarget in motor function, (c) included a larger set of important studies (27, 28, 32, 34, 36, 40, 51) that bring relevant approaches to the field under investigation and that were published after previous reviews were carried out, and (d) analyzed the association of certain therapeutic variables with tDCS effect size. The recent evidence-based guidelines for neuromodulation target sites for the treatment of motor function in PD concludes that anodal tDCS over motor, premotor, and supplementary motor area is likely to be effective (level C), whereas on the prefrontal cortex, there is possibly no efficacy (level B) (15). Thus, considering the gaps that still exist in the literature and seeking clarification in future recommendations, further studies should include secret allocation, adequate blinding, homogeneous comparison group, sufficient sample size, application of tDCS in single and multiple brain regions, shorter interval of hours between sessions, and evaluation of the long-term effect on simple and complex motor tasks. Finally, future studies could go beyond the target area and investigate patterns of cortical activation at baseline and during treatment to infer possible predictors of response to therapy. A deeper look at the neurophysiological correlates in patients with PD is needed, particularly to provide neurophysiological evidence that cholinergic dysfunction may be an important and early contributor to motor and cognitive dysfunction in PD.

## Conclusion

In summary, this systematic review with meta-analysis and meta-regression found no significant short-term effect of tDCS alone on motor function, balance, gait, dyskinesia, and motor fluctuations, regardless of brain area or number of stimulated nominal targets in patients with PD. We also found no relationship between the effect of tDCS alone and the number of sessions or cumulative treatment time.

## Data Availability Statement

The data supporting the conclusions of this review article will be made available by the corresponding author on request to qualified researcher.

## Ethics Statement

Ethical review and approval was not required for the study on human participants in accordance with the local legislation and institutional requirements. Written informed consent for participation was not required for this study in accordance with the national legislation and the institutional requirements.

## Author Contributions

PO and TA contributed with preliminary databases' searches, selection of studies, screening of research results and eligibility criteria, data extraction, risk of bias, quality assessment, data analysis, and manuscript writing. DM supervised the systematic review of studies, reviewed, and edited the manuscript. AR contributed to the meta-regression analysis. MB, SA, AO, HS, RP, and EM reviewed and edited the manuscript. All authors listed above have made a substantial, direct and intellectual contribution to this work, contributed to manuscript revision, read, and approved the submitted version.

## Funding

This study had neither direct nor specific funding, but was financed in part by: Live Without Limits Brazilian Programme for Disabilities/Ministry of Health (Brazil)—Portaria N 2.164/GM/MS: funds the operation and pays professionals' salaries of the Rehabilitation Center/Anita Garibaldi Center for Education and Health/Santos Dumont Institute. Coordenação de Aperfeiçoamento de Pessoal de Nível Superior—Brazil (CAPES)/Ministry of Education (Brazil)—Finance Code 001: funds postgraduate scholarships in neuromodulation at Edmond and Lily Safra International Institute of Neuroscience/Santos Dumont Institute. AHO financial support from CNPq (487361/2013-0 and 479000/2012-3), FAPESP (13/10187-0 and 14/101347). EM and AHO financial support from FAPESP/CEPID/BRAINN (13/07559-3). Santos Dumont Institute—Management Contract by Ministry of Education (Brazil): funds the operation and pays Researcher's salaries of Santos Dumont Institute.

## Conflict of Interest

The City University of New York holds patents on brain stimulation with MB as inventor. MB has equity in Soterix Medical Inc. MB consults, received grants, assigned inventions, and/or serves on the SAB of SafeToddles, Boston Scientific, GlaxoSmithKline, Biovisics, Mecta, Lumenis, Halo Neuroscience, Google-X, i-Lumen, Humm, Allergan (Abbvie), Apple. HS served as a consultant for Abbott, LivaNova, and Boston Scientific in the previous 5 years. The remaining authors declare that the research was conducted in the absence of any commercial or financial relationships that could be a potential conflict of interest.

## Publisher's Note

All claims expressed in this article are solely those of the authors and do not necessarily represent those of their affiliated organizations, or those of the publisher, the editors and the reviewers. Any product that may be evaluated in this article, or claim that may be made by its manufacturer, is not guaranteed or endorsed by the publisher.

## Abbreviations

PD: Parkinson's disease
tDCS: transcranial direct current stimulation
a-tDCS: anodic transcranial direct current stimulation
c-tDCS: cathodic transcranial direct current stimulation
DALY: Disability-adjusted life year
PRISMA: Preferred Reporting Items for Systematic Reviews and Meta-Analyses
PEDro: Physiotherapy Evidence Database
UPDRS: Unified Parkinson's Disease Rating Scale
HY: Hoehn and Yahr
FOG: Freezing of gait
MD: Mean difference
SMD: Standardized mean difference
CI: Confidence interval
DLPFC: Dorsolateral prefrontal cortex
M1: Primary motor cortex
TUG: Timed up and go
BBS: Berg Balance Scale.
